# New Variations on the Theme of Multidimensional Geriatric Assessment

**DOI:** 10.3390/geriatrics5040104

**Published:** 2020-12-17

**Authors:** G. Darryl Wieland

**Affiliations:** Duke University Center for the Study of Aging and Human Development, Durham, NC 27708, USA; gdw9@duke.edu

Geriatric assessment—broadly defined—has become foundational to systems of care for frail elderly people at risk for functional decline, death, intensification of services, and long-term institutionalization. Its key feature is the ascertainment of multiple dimensions of health and health risks: not only medical, but functional, cognitive, psychological, and socioeconomic factors. This multidimensionality is key to systematic screening and targeting using technologies to uncover frail, at-risk elderly people in their neighborhoods, homes, and at various other service contact points, for more intensive evaluation, i.e., “comprehensive geriatric assessment”, a multidisciplinary diagnostic and treatment process that identifies medical, psychosocial, and functional limitations of a frail older person to develop a coordinated plan to maximize overall health with aging [1]. 

Geriatric care models embedding comprehensive and multidimensional assessment—in the community and in institutions—have been studied for years, with evidence supporting the efficacy of some in improving various outcomes [2,3,4]. In fact, early successes are partly responsible for the spread and differentiation of assessment-based programs, involving teams of specially trained health professions, together with the continued growth in the number of frail and at-risk elderly people in demographically post-transition populations. This has been observed of geriatric assessment in other journal collections going back some decades [5,6]. From those earlier days, multidimensional geriatric assessment has come to support a variety of “co-care” or collaborative approaches with orthopedics, oncology, emergency medicine, surgery, and other medical disciplines. Now, developing countries are also rapidly aging and becoming wealthier, with improved health and social service resources. Thus, the interest in geriatric medicine and the related care system has been spreading, as well as the need to adapt and evaluate practice and assessment technologies in these new environments.

The papers gathered in this Special Issue of *Geriatrics* all relate in some way to the foundational theme of multidimensional geriatric assessment, as they also exhibit the continuing evolution and differentiation of structures and processes of care built upon it. Implemented or anticipated assessment-based models of “co-care” with other specialties, allied health providers, or for special clinical populations, are the subject of several reports [7,8,9,10]. One offering describes a protocol for a new experimental trial of comprehensive geriatric assessment in an acute care unit [11]; as person-centeredness is a common fundamental concern of clinicians and teams performing such assessments, it is fitting to have a second qualitative study on this topic from the same group [12]. Attention to common geriatric syndromes (e.g., falls) and assessment in developing countries and in multicultural immigrant populations is demonstrated in two other reports [13,14]. The use of technologies to more precisely assess the aspects of health of older patients toward the improvement of their care has been burgeoning in recent years, and is exemplified here by one contribution [15]. Finally, of great concern is the relative paucity of geriatrics professionals globally, weighed against the increasing need for them nearly everywhere. Numerous strategies have arisen to deal with these workforce and educational limitations, two of which are raised by the final papers in this Special Issue. First is the use of widespread screening and targeting by non-geriatrics professionals to identify older persons for further clinical assessment and associated care; the issue of how well these processes work is treated in the papers from Young and Smithard, and Broad et al. [16,17]. Buhr et al. evaluate the efficacy of direct transfers of geriatric assessment knowledge and skills from model geriatric resources teams to primary care practices [18].

Undoubtably, the papers appearing here represent only a small sampling of present activity, but they indicate the ongoing relevance of foundational principles as services and care for aging populations expand. New technologies, populations, systems of care and financing, and workforce development strategies will need to hold true to these core principles. 
